# Mentalizing, Loneliness and Pain-Related Depressive Symptoms Are Associated with Pain Severity in Patients with Rheumatic Diseases: Results of a Cross-Sectional Secondary Analysis

**DOI:** 10.3390/jcm14113624

**Published:** 2025-05-22

**Authors:** David Riedl, Julia Karnik, Astrid Lampe, Christina Kirchhoff, Karin Labek, Michael Schirmer

**Affiliations:** 1 University Hospital of Psychiatry II, Department of Psychiatry, Psychotherapy Psychosomatics and Medical Psychology, Medical University of Innsbruck, 6020 Innsbruck, Austria; 2 Ludwig Boltzmann Institute for Rehabilitation Research, 1140 Vienna, Austria; 3 Department of Internal Medicine, University Clinic II, Medical University of Innsbruck, 6020 Innsbruck, Austria; 4 Rehabilitation Center Montafon, 6780 Schruns, Austria; 5 Institute of Psychology, University of Innsbruck, 6020 Innsbruck, Austria

**Keywords:** mentalization, psychosomatics, mediation analysis, pain, depression, loneliness, rheumatic disease

## Abstract

**Background/Objectives**: Patients with rheumatic diseases often experience pain-related depressive symptoms, potentially exacerbated by feelings of loneliness and social isolation. This study explores the role of mentalizing, i.e., the understanding of inner mental states in oneself and others, as a protective factor in this context. **Methods**: In this secondary analysis, *n* = 76 patients completed the FESV depression scale, MZQ UCLA loneliness scale and pain severity items from the German Pain Questionnaire. Structural equation models and mediation analyses were employed to test different theoretical models. **Results**: The best model fit was found for Model 3, which described the association of loneliness with pain severity (β = 0.34, *p* = 0.004). The association was fully mediated by a sequential mediation of mentalizing and pain-related depression. Adding the mediators increased the overall explained variance of pain severity from 12% to 41% with an excellent model fit (CFI > 0.99; TLI > 0.99; RMSEA = 0.001). **Conclusions**: The study suggests that patients’ pain severity may be influenced by the interaction between loneliness, depressive symptoms and mentalizing abilities. The negative impact of pain-related depressive symptoms and loneliness on pain severity underscores the need for their targeted management in routine care for chronic pain patients. Improvement of mentalizing may be a resilience factor for these patients.

## 1. Introduction

Pain is a prominent symptom in the majority of patients with rheumatic conditions, particularly in immune-mediated chronic diseases such as rheumatoid arthritis (RA), axial spondyloarthritis (axSpA), peripheral spondyloarthritis (pSpA) including psoriatic arthritis (PsA), vasculitides, and other collagenoses. Elevated pain levels are often accompanied by diminished physical, psychological, and social functioning [1], resulting in a significant reduction in the patients’ quality of life. Overall, this represents a major burden associated with rheumatic diseases [2,3]. In patients experiencing chronic pain, it is common to observe psychological comorbidities such as feelings of sadness, tearfulness, emptiness, hopelessness, anger, irritability, or frustration. While inflammation-related biological factors may explain this association in patients with RA to some extent [4], it often persists even in patients with only low disease activity, suggesting that psychological factors also play a significant role.

Depression and catastrophic thoughts have been linked to heightened levels of pain and poorer pain coping in patients with rheumatic diseases [5], and an extensive body of literature has described the bidirectional relationship between pain and depression [6,7]. One common risk factor frequently associated with both pain and depression is a sense of loneliness, characterized by a socially painful state of perceived social isolation [8,9,10,11]. Studies have demonstrated that individuals who experience social isolation tend to have a significantly lower pain tolerance compared to those who feel socially connected [12]. Higher levels of loneliness are frequently associated with later levels of depression [13,14] and acute or chronic pain [15]. It has been suggested that common brain mechanisms underlie both physical and social pain [16,17] as well as immunometabolic pathologies [18], thereby establishing a connection between loneliness and elevated levels of pain and depression [18,19]. It has been argued that patients with pain-related depression may be more likely to report somatic symptoms such as fatigue, sleep disturbances or physical pain rather than more cognitive symptoms such as persistent sadness, anhedonia, and feelings of worthlessness [20].

However, the experience of loneliness cannot be fully explained by objective indicators of isolation, such as the number of social contacts [21]. It is presumed that satisfaction with social relationships and the sense of social isolation are influenced by early childhood experiences related to meeting the need for closeness and tenderness or the availability of attachment figures [22]. In cases where attachment experiences are disrupted during childhood, children may develop deficiencies in their mentalizing capabilities [23], which are defined as mental processes that allow individuals to understand and represent inner mental states in themselves and others, which includes considering one’s own thoughts, needs, emotions, wishes, and desires, as well as those of others [24,25]. Deficits in mentalization can manifest as a lack of emotional awareness and self-reflection, equating inner mental states with external reality [26]. Consequently, these deficits are closely linked to an individual’s social functioning and their feelings of social inclusion or isolation.

Deficits in mentalizing have been identified as a significant risk factor in various mental health disorders. Specifically designed treatment approaches, such as Mentalization-Based Treatment (MBT) [27], have been developed and proven to substantially enhance the mental health, overall functioning, and vocational status of patients with Borderline Personality Disorder (BPD), demonstrating long-term effectiveness [28,29]. In recent years, the treatment concepts to improve mentalization were adapted for a broad range of other disorders, including depression [30,31,32,33], posttraumatic stress disorder [34], dissociation [35], and eating disorders [23,36,37]. In a recent study, we found that improvements in mentalization played a crucial role in the process of change during psychosomatic rehabilitation treatment. These improvements were closely linked to enhanced social functioning and increased social participation [38].

In summary, patients with rheumatoid arthritis (RA) often experience comorbid pain-related depression, which may be partly attributed to feelings of social exclusion and loneliness. Elevated levels of depression are associated with more severe pain experiences and pain severity. An underlying protective factor may involve enhancing the patients’ mentalizing capabilities, which could foster social integration and, in turn, reduce the level of loneliness, depression and pain experienced by patients. However, empirical studies have not yet established this relationship.

The objective of this study was to examine the association between mentalizing, loneliness, pain-related depressive symptoms and pain severity in a sample of chronic pain patients. Since the nature of the available data does not allow for causal interpretation, we have tested different models to identify potential pathways for the association of these variables. We generally hypothesized that (a) mentalizing, loneliness, pain-related depressive symptoms and pain severity are significantly correlated factors; (b) higher levels of mentalizing are associated with lower pain severity; and (c) heightened levels of loneliness and pain-related depressive symptoms are associated with higher pain severity.

## 2. Materials and Methods

### 2.1. Patients and Procedures

This is a secondary analysis of data collected in a prospective, double-blinded, randomized, and controlled study at a rheumatology outpatient clinic [39]. Patients were randomized to wearing colored wristbands as a non-verbal communication tool, or they received general disease and treatment information. Patients completed questionnaires at study entry (T1) and at the following visit as the end of the study (T2) after 4.5 months. Out of 102 eligible patients approached during the recruitment period, 87 consented and completed baseline assessments, and 76 were included in the analysis due to complete data. This sample size, while modest, reflects the exploratory nature of this secondary analysis and was sufficient for SEM with bootstrapping as a robustness method. Since the control arm of the study was too small to conduct longitudinal analyses, only baseline data (T1) was analyzed in the present study. Inclusion criteria were (a) age > 18 years, (b) no apparent cognitive impairment, and (c) confirmed diagnosis of a rheumatic disease. Exclusion criteria were (a) visual loss or color blindness, (b) severe psychiatric diseases, and (c) silicone allergy. Diagnoses were defined by rheumatologists based on currently used classification criteria if available.

The study protocol was approved by the ethics committee of the Medical University of Innsbruck on 21 July 2021 (AN 1219/2021). The study was performed according to the Helsinki criteria, and patients were recruited only after informed and written consent. Data were pseudonymized for statistical analyses and anonymized prior to publication. Patients did not receive any payment or other kinds of rewards for participation in the study.

### 2.2. Measures

#### 2.2.1. Mentalization Questionnaire (MZQ)

The ability for mentalizing was assessed using the German Version of the Mentalization Questionnaire (MZQ). The original version of the MZQ was developed as a self-rated instrument to assess mentalizing from a patient’s perspective [40]. It consists of 15 items with responses ranging from ‘no agreement at all’ to ‘total agreement’ on a 5-point Likert scale. The total score can range between 15 and 75. In this study, we have reversed the MZQ scores, thus higher scores indicate better mentalizing. In a recent validation in the German general population, good reliability and validity were reported for the MZQ total score [41]. In our sample, a good internal consistency was also found for the MZQ total score (ω = 0.88).

#### 2.2.2. Pain Related Depressive Symptoms

Pain-related depressive symptoms were assessed with the depression subscale of the German pain-coping questionnaire FESV (German: Fragebogen zur Erfassung der Schmerzverarbeitung) [42]. The subscale consists of five that assess pain-related feelings of depression (e.g., Item 3, ‘*Because of my pain I often feel sad*.’). Responses range from 1 ‘total disagreement’ to 6 ‘total agreement’, with higher scores indicating worse pain-related depression. Good psychometric properties were reported for the FESV [42]. In our sample, excellent internal consistency was observed (ω = 0.91).

#### 2.2.3. UCLA Loneliness Scale

Patients subjective feelings of loneliness as well as feelings of social isolation was assessed with the UCLA Loneliness Scale [43]. This scale consists of 20 items that are scored on a 5-point Likert scale (1 = never, 5 = always), with higher total scores reflecting higher levels of loneliness. The German validation of the UCLA Loneliness Scale revealed good psychometric properties and a three-factor structure, namely loneliness, emotional isolation and social isolation [44]. In our sample, good internal consistency was found for the total score of the UCLA Loneliness Scale (ω = 0.92) and good internal consistency for the subscales loneliness (ω = 0.82), emotional isolation (ω = 0.83) and social isolation (ω = 0.83).

#### 2.2.4. Pain Severity

To assess the level of pain severity, patients were asked to rate three items regarding their level of pain severity on a numeric rating scale (NRS) from the German Pain Questionnaire, namely pain-related impairment of working ability, leisure activities, and activities of daily living (ADL) during the last three months [45]. The scales ranged from 0 (no impairment) to 10 (complete impairment). A mean score was calculated for the three impairment items to generate an overall pain severity score, ranging from 0 to 10. An excellent internal consistency between the three impairment items was observed in our sample (ω = 0.92).

#### 2.2.5. Clinical Data

The case report form included data on age, sex, time of first symptom, diagnosis and laboratory data. Clinical assessment of disease activity was performed in patients with RA, PsA and axial SpA using the Clinical Disease Activity Index (CDAI), the Clinical Disease Activity in Psoriatic Arthritis Score (cDAPSA) and the Bath Ankylosing Spondylitis Disease Activity Index (BASDAI), respectively. Laboratory parameters like levels of C-reactive protein (CRP), erythrocyte sedimentation rate (ESR) were assessed at study entry.

### 2.3. Statistical Procedures

Patients with complete datasets for the MZQ, FESV and pain severity scale were included in the analyses. In line with Boonstra et al. [46], the NRS pain impairment ratings were grouped as 0 as ‘no impairment’, 1–5 as ‘mild impairment’ and ≥6 as ‘moderate to severe impairment’. Differences in mentalizing, pain-related depressive symptoms, and pain severity between diagnostic groups and sex were investigated using analyses of variance (ANOVAs) and independent sample *t*-tests. Associations between baseline scores for mentalizing, pain-related depressive symptoms, and pain severity were investigated by calculation of Pearson correlation coefficients.

**Figure 1:**
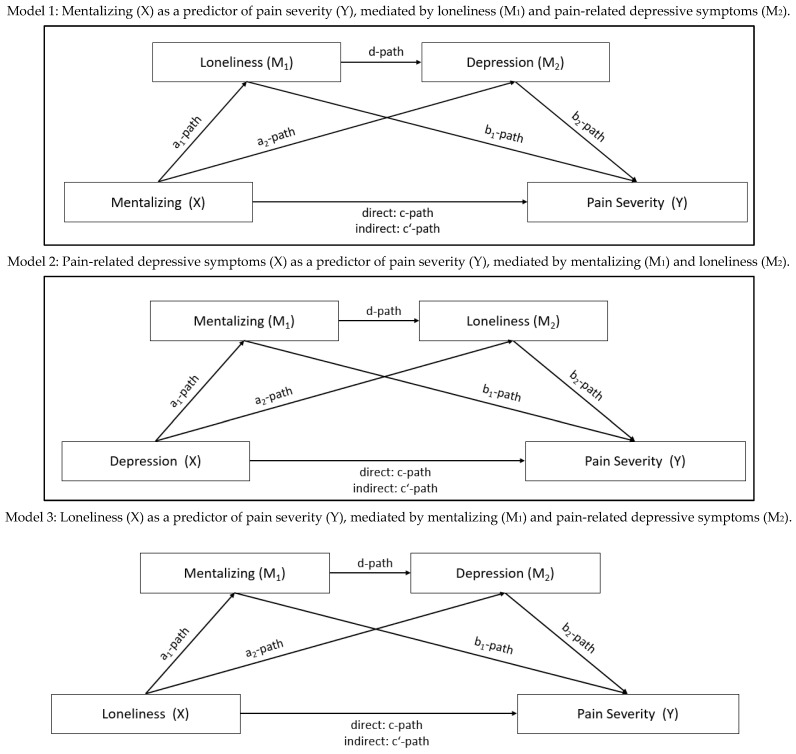
Theoretical model of the interaction of mentalizing, loneliness, pain-related depressive symptoms and pain severity.

The association of mentalizing, loneliness, pain-related depressive symptoms, and pain severity was analyzed using structural equation modeling (SEM). Due to the cross-sectional and non-interventional design, no definite causal interpretation was possible. Therefore, different models were tested to identify potential pathways of the association of mentalizing, loneliness, pain-related depressive symptoms and pain severity. In Model 1, mentalizing was defined as a predictor for pain severity and loneliness, and depressive symptoms were added as mediators of this association in a sequential pattern. In Model 2, pain-related depressive symptoms were defined as the predictor for pain severity, and mentalizing and loneliness were then added as mediators to the model. Finally, in Model 3, loneliness was defined as a predictor for pain severity, and mentalizing and depressive symptoms were added as mediators. The theoretical models are shown in Figure 1.

Bootstrapped confidence intervals (5000 samples, 95% CI) were calculated to evaluate the statistical significance of all included paths in the SEM, accounting for non-normal distribution of data due to the small sample size. The Bollen–Stine bootstrapping procedure was additionally used to evaluate model fit under non-normality assumptions, supporting the robustness of the SEM results. To determine the model’s goodness of fit, Pearson’s chi-squared test (χ^2^) with degrees of freedom, the comparative fit index (CFI), the Tucker–Lewis Index (TLI) and the root mean square error of approximation (RMSEA) with lower and higher bounds of the 95% confidence interval (CI) were calculated. To evaluate whether the empirical data were closely fitting the theoretical model, the *p*-value of Close Fit (PCLOSE) was calculated based on the RMSEA values, with values of *p* > 0.05 indicating close fit and *p* < 0.05 indicating worse than close model fit. Acceptable goodness of fit was defined as RMSEA values of <0.08 and CFI/TLI values > 0.90 [47]. *p*-values < 0.05 (two-sided) were considered statistically significant. Bollen–Stine values > 0.05, indicating a good fit. Statistical analyses were performed with IBM SPSS (v22.0) and SPSS AMOS (v24.0).

## 3. Results

The initial sample with completed questionnaires at T1 consisted of 87 patients. Due to missing questionnaire data at T2, *n* = 11 patients were excluded from the analyses. The remaining 76 patients showed a mean age of 45.6 years; the majority of patients were female (*n* = 61, 80.3%). Approximately half of the sample was obese (*n* = 39, 51.3%), and the most frequent diagnoses were RA (*n* = 25, 32.9%) and axial SpA (*n* = 24, 31.6%). Characteristics of included patients are detailed in Table 1.

There was no significant difference in mentalizing (F(3, 73) = 88.09, *p* = 0.52) or pain-related depressive symptoms (F(3, 73) = 80.26, *p* = 0.15) between patients with different diagnoses. Neither for loneliness nor pain severity was there a significant difference between patients with different diagnoses (*p* = 0.27–0.85). Gender differences were not observed for mentalizing, pain-related depressive symptoms, loneliness, or pain severity (*p* = 0.06–0.97).

### 3.1. Baseline Association of Loneliness, Pain-Related Depressive Symptoms, Pain Severity and Mentalizing

**Figure 2:**
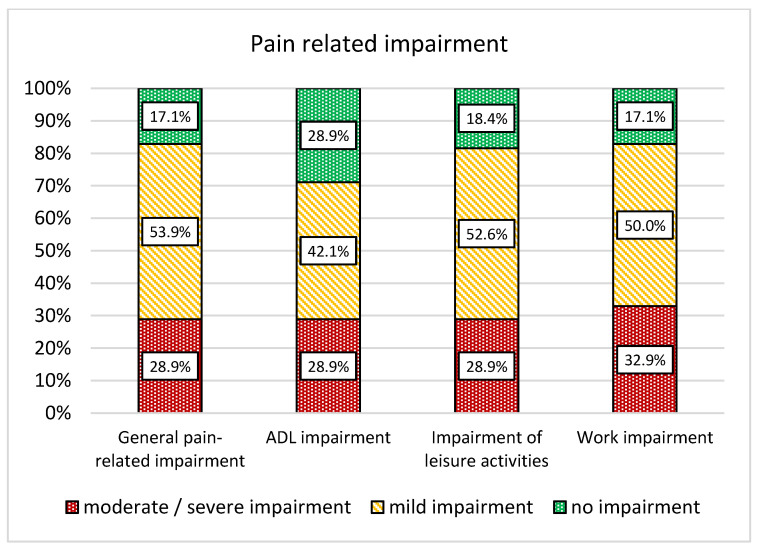
Severity of general pain severity, working impairment, as well as pain severity of leisure activities and activities of daily living (ADL).

At baseline, 82.8% (*n* = 63) of the patients reported at least mild pain severity, with 28.9% (*n* = 22) reporting moderate to severe pain. Rates for impairments of leisure activities and work impairment were comparable, while patients reported slightly lower ADL impairment. For details, see Figure 2.

As shown in Table 2, higher levels of mentalizing capabilities at baseline were associated with lower levels of loneliness and pain-related depressive symptoms, as well as lower impairment of ADL, leisure time activities, and working ability. Loneliness was associated with more pain-related depressive symptoms and ADL, leisure time activities, and working ability. Higher pain-related depressive symptoms were further correlated with more ADL, leisure time activities, and working ability. For details, see Table 2.

### 3.2. The Association of Mentalizing, Loneliness, Pain-Related Depressive Symptoms and Pain Severity

**Figure 3:**
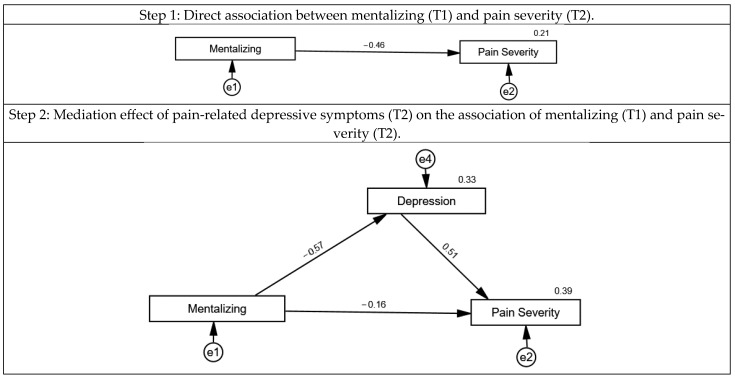

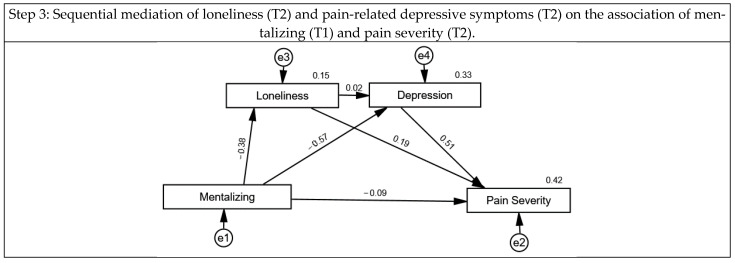
Structural equation model of the association of mentalizing with pain severity, mediated by loneliness and pain-related depressive symptoms in a sample of *n* = 76 chronic pain patients. Circles represent error terms (e). Numbers next to arrows in the model represent standardized estimates, and numbers next to factors represent the R^2^, i.e., the explained variance.

In Model 1 it was assumed that mentalizing would predict the level of pain severity and that this association was mediated by feelings of loneliness and depressive symptoms, see Figure 3. In a first step, the direct association of mentalizing and pain severity was tested. Higher mentalizing was associated with lower pain severity (β = −0.46, 95% CI: −0.64–−0.23; *p* < 0.001) and explained 21% of its variance. In a second step, pain-related depressive symptoms were added to the model. Lower mentalizing predicted higher pain-related depressive symptoms (β = −0.57, 95% CI: −0.72–−0.37; *p* = 0.001) and explained 33% of its variance. Higher pain-related depressive symptoms were significantly associated with more pain severity (β = 0.52, 95% CI: 0.28–0.68; *p* = 0.001). The direct association between mentalizing and pain severity was no longer statistically significant after adding pain-related depressive symptoms to the model (β = −0.16, 95% CI: −0.37–0.06; *p* = 0.14), and the overall explained variance increased to 39%, thus indicating a full mediation. In a third step, feelings of loneliness were added to the model. Lower mentalizing was associated with higher feelings of loneliness (β = −0.38, 95% CI: −0.58–−0.15; *p* < 0.001) and predicted 15% of its variance. Higher levels of loneliness were neither significantly associated with higher pain-related depressive symptoms (β = 0.02, 95% CI: −0.19–0.23; *p* = 0.90) nor with pain severity (β = 0.19, 95% CI: −0.05–0.42; *p* = 0.13). Acceptable to good model fit was found for the final model after exclusion of non-significant paths, except for the RMSEA (*χ*^2^ (2) = 3.936, *p* = 0.14; CFI = 0.98; TLI = 0.93; RMSEA = 0.114, 95% CI: 0.000–0.280; PCLOSE = 19; Bollen–Stine bootstrap: *p* = 0.21).

**Figure 4:**
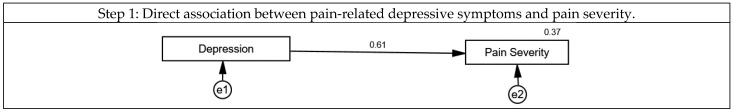

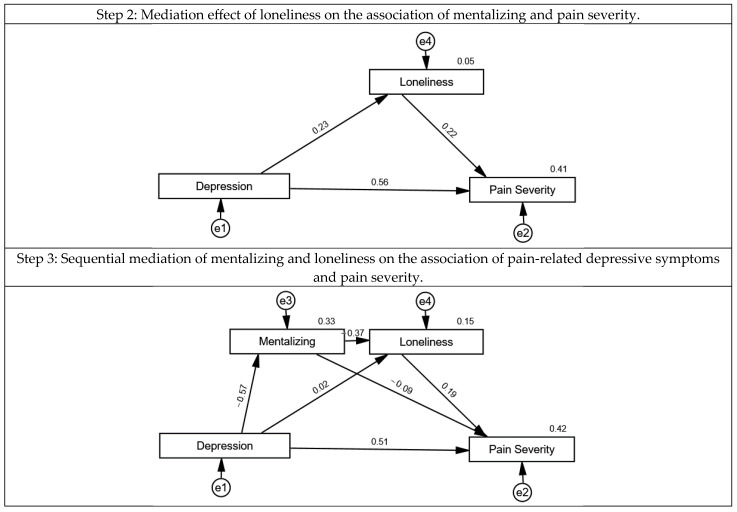
Structural equation model of the association of pain-related depressive symptoms with pain severity, mediated by mentalizing and loneliness in a sample of *n* = 76 chronic pain patients. Circles represent error terms (e). Numbers next to arrows in the model represent standardized estimates, and numbers next to factors represent the R^2^, i.e., the explained variance.

In Model 2, it was assumed that pain-related depressive symptoms would predict the level of pain severity and that this association was mediated by feelings of loneliness and mentalizing capabilities, see Figure 4. In a first step, the direct association of depressive symptoms and pain severity was tested. Higher pain-related depressive symptoms were associated with higher pain severity (β = 0.61, 95% CI: 0.42–0.76; *p* < 0.001) and explained 37% of its variance. In a second step, loneliness was added to the model. However, pain-related depressive symptoms did not statistically significantly predict loneliness (β = 0.23, 95% CI: −0.01–0.45; *p* = 0.06), and neither was loneliness directly associated with pain severity (β = 0.22, 95% CI: −0.01–0.43; *p* = 0.052). The direct association between pain-related depressive symptoms and pain severity remained statistically significant (β = 0.56, 95% CI: 0.37–0.72; *p* = 0.001), and the overall explained variance slightly increased to 41%. In a third step, mentalizing was added as an additional predictor to the model. Lower mentalizing was associated with higher pain-related depressive symptoms scores (β = −0.57, 95% CI: −0.72–−0.37; *p* = 0.001) and predicted 33% of its variance. Lower levels of mentalizing were further associated with higher loneliness (β = −0.37, 95% CI: −0.61–−0.08; *p* = 0.011), but not with pain severity (β = −0.09, 95% CI: −0.31–0.15; *p* = 0.46). Acceptable to good model fit was found for the final model after exclusion of non-significant paths, except for the RMSEA (*χ*^2^ (2) = 6.034, *p* = 0.11; CFI = 0.96; TLI = 0.92; RMSEA = 0.116, 95% CI: 0.000–0.251; PCLOSE = 17; Bollen–Stine bootstrap: *p* = 0.18).

**Figure 5:**
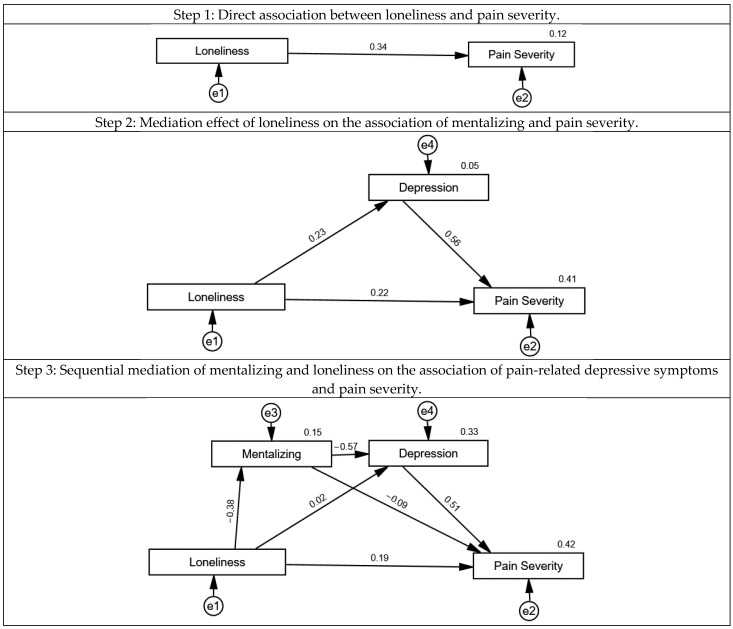
Structural equation model of the association of loneliness with pain severity, mediated by mentalizing and pain-related depressive symptoms in a sample of *n* = 76 chronic pain patients. Circles represent error terms (e). Numbers next to arrows in the model represent standardized estimates, and numbers next to factors represent the R^2^, i.e., the explained variance.

Finally, in model 3, it was assumed that loneliness would predict pain severity and that this association was influenced by levels of pain-related depressive symptoms and mentalizing, see Figure 5. In a first step, the direct association of loneliness and pain severity was tested. Higher levels of loneliness were associated with higher pain severity (β = 0.34, 95% CI: 0.11–0.55; *p* = 0.004) and explained 12% of its variance. In a second step, depressive symptoms were added to the model. While higher loneliness was not directly associated with higher pain-related depressive symptoms (β = 0.23, 95% CI: −0.01–0.45; *p* = 0.06), higher pain-related depressive symptoms were significantly associated with more pain severity (β = 0.56, 95% CI: 0.37–0.72; *p* = 0.001), and the direct association between mentalizing and pain severity was no longer statistically significant (β = 0.22, 95% CI: −0.01–0.43; *p* = 0.052). The overall explained variance substantially increased to 41%. While this value reflects a moderate-to-large effect in social and clinical research contexts, it also suggests that these psychosocial factors account for a substantial proportion of variance in patients’ pain experiences. Clinically, this supports the importance of targeting mentalizing and depression in pain management.

In a third step, mentalizing was added to the model. Higher loneliness was associated with lower mentalizing (β = −0.38, 95% CI: −0.58–−0.15; *p* < 0.001) and predicted 15% of its variance. Higher mentalizing was significantly associated with higher pain-related depressive symptoms scores (β = −0.57, 95% CI: −0.73–−0.35; *p* < 0.001), but not directly with pain severity (β = −0.09, 95% CI: −0.31–0.15; *p* = 0.46). Excellent model fit was found for the final model after exclusion of non-significant paths (*χ*^2^ (2) = 0.651, *p* = 0.72; CFI > 0.99; TLI > 0.99; RMSEA = 0.001, 95% CI: 0.000–0.164; PCLOSE = 76; Bollen–Stine bootstrap: *p* = 0.74).

## 4. Discussion

The objective of this study was to investigate possible associations of mentalizing abilities, depressive symptoms and feelings of loneliness with levels of pain severity in patients with rheumatic diseases.

In this study, baseline data collected for a randomized controlled trial regarding a non-verbal communication tool in patients with rheumatic diseases was analyzed. In our sample, nearly one-third of the patients reported moderate to severe pain severity, especially in terms of work-related impairment. The high prevalence of work-related pain impairment aligns with international studies, such as Sokka et al. [48]: while upon first experiencing symptoms, a majority of men (ranging from 57% to 100% among countries) and women (ranging from 19% to 87%) below the age of 65 years were employed, a considerable portion of these individuals (37%) reported subsequent work disability due to RA in the course of the disease. However, the same study also showed that patients who continued to work had better clinical status across all measures and self-reported scores, with similar patterns observed across countries with high and low gross domestic products.

As we hypothesized, there was a significant cross-sectional association between loneliness, pain-related depressive symptoms, and pain severity in our sample, which aligns with previous studies [8,9,10,11,13]. Additionally, we discovered that higher levels of mentalizing, which refers to the ability to understand and represent inner mental states of oneself and others, were linked to lower levels of loneliness and pain-related depressive symptoms. This finding is consistent with recent research as well [49]. It has been suggested that individuals with elevated levels of depression may exhibit poorer cognitive and affective mentalizing skills, including difficulties in emotion recognition (i.e., decoding tasks) and identifying the intentions of others (i.e., reasoning tasks) [50,51]. This could be due to a negative evaluation bias that leads depressed individuals to infer negative mental states [52,53]. Furthermore, our study revealed that lower mentalizing abilities were associated with more severe pain severity. Patients with chronic pain often find it challenging to regulate their emotions, resulting in less frequent use of emotional regulation strategies, which in turn can lead to the development of dysregulated emotions [54], and thus pain can be understood as an expression of these dysregulated or non-mentalized emotions [55].

While our correlation analyses suggested an association between mentalizing, pain-related depressive symptoms, loneliness, and pain severity, the nature of this relationship remained unclear. To further investigate this assumption, we tested three different theoretical models that explored the relationship between mentalizing and pain severity, as well as the potential mediating roles of loneliness and pain-related depressive symptoms. In the first theoretical model, we hypothesized that mentalizing would predict pain severity, with this association mediated by feelings of pain-related depressive symptoms and loneliness. The results supported the assumption that mentalizing is a predictor of pain severity. However, loneliness did not emerge as a significant predictor of either pain-related depressive symptoms or pain severity. Instead, pain-related depressive symptoms fully mediated the relationship between mentalizing and pain severity. Although the model fit was generally acceptable, RMSEA metrics indicated a poor fit to the data.

In our second model, we posited that depressive symptoms would predict pain severity, with loneliness acting as a mediator influenced by patients’ mentalizing capabilities. However, the results of the structural equation model did not support this hypothesis. Neither loneliness nor mentalizing was identified as a mediator in the relationship between pain-related depressive symptoms and pain severity. A possible explanation is that loneliness may not operate as a proximal cause of physical pain but rather exerts its effect through emotional dysregulation, manifesting as depressive symptomatology. These findings align with biopsychosocial models of pain, which emphasize the role of psychological distress—such as depression—as a more immediate contributor to pain perception. Additionally, it is conceivable that the subjective experience of loneliness intensifies negative affect, thereby increasing vulnerability to depressive symptoms, which in turn exacerbate perceived pain.

In the third theoretical model, we hypothesized that patients experiencing higher levels of loneliness would report greater pain severity. We further assumed that depressive symptoms would mediate this relationship and would themselves be predicted by the patients’ capacity for mentalizing, which in turn would be influenced by loneliness. Our findings largely supported this model. Patients who reported higher levels of loneliness also reported greater pain severity. However, when depressive symptoms were added to the model, the direct association between loneliness and pain severity was no longer statistically significant. Loneliness was, however, directly associated with depressive symptoms—an important finding. Additionally, our data showed that loneliness significantly predicted lower mentalizing abilities, which in turn predicted higher levels of depressive symptoms. These symptoms were significantly associated with increased pain severity. We found excellent fit indices for the third theoretical model, thus indicating that the model was well in line with the empirical data in our study.

In summary, the lonelier a patient feels, the less capable they are of understanding their own internal experiences as well as those of others (i.e., mentalizing). Reduced mentalizing abilities are associated with greater depressive symptoms, which in turn are linked to more severe pain impairment. We therefore assume that the ability to mentalize, which involves recognizing and understanding one’s own physical and mental states, could facilitate the processing of unpleasant experiences, effectively enabling individuals to cope with emotional distress by thoroughly addressing it, rather than resorting to other, less functional coping mechanisms. Thus, improvement in patients’ mentalizing capabilities can be considered as a critical success factor in the psychosomatic treatment of patients with chronic physical and mental health disorders, which is in line with previous studies [35,38,56,57]. It has been suggested that the process of suffering from physical diseases may be influenced by the level of insight and self-awareness [58]. It has been further described in previous studies that pain perception is strongly associated with emotions such as depression, anger and grief [59]. Our results further underscore the importance of feelings of social isolation and loneliness when referring to a patient’s mentalizing capability, which is in line with previous research [60].

Thus, mentalization-focused patient–physician interactions may help to improve practical pain management [61]. Previous studies have shown that the patient-clinician relationship directly influenced adherence to treatment protocols as well as treatment outcomes [62,63,64]. A physician who validates the patients’ pain by acknowledging the experience and demonstrating belief in it by empathically responding to the patient shows that the physician is mentalizing the patient’s experience. By doing so, it is communicated to the patient, that the patient’s experience is worthy of active consideration and thus enables the patient to discuss the pain and difficulties without fear of reprisal or judgment [65], which facilitates the patient’s trust in the therapeutic relationship. Patients can additionally improve their mentalizing capabilities by psychotherapeutic techniques, such as mindfulness meditation. This technique enhances the patients’ awareness of their thoughts and emotions, thereby promoting the development of mentalizing abilities, reducing pain and improving overall well-being [66,67]. Psychoeducation on the role of mentalizing in emotional regulation and the consequences of impaired mentalizing may encourage patients to adopt a more reflective stance toward their experiences. Additionally, simple decentering exercises, such as compassionate writing exercises [68], may help to have ‘one’s mind in mind’. We therefore believe that the development of mentalization-oriented manuals for practitioners of different professions could further improve the care of chronic pain patients in the future and that more research in this field is warranted and needed.

The strength of the study is certainly the new concept of integrating mentalization into the reduced physical and mental functioning of patients with rheumatic diseases. The main limitation, however, is that this study was designed as a pilot study to test a social intervention and not primarily to test the possible influence of mentalization on patients with rheumatic diseases. We therefore decided to only use cross-sectional baseline data to avoid any bias in interpretation. The relatively small sample size (*n* = 76) restricts the statistical power and may limit generalizability. Larger and more diverse samples are needed in future research to validate these preliminary findings across different demographic and clinical subgroups. Nevertheless, the results are promising and show that further studying the interplay between all three components of health (namely, physical, psychological and social functioning) will provide further evidence on how to improve psychological and social interventions in these patients. Then, the main limitation of the study can be overcome, that at the moment age- and sex-dependent comparisons of the model between different diagnostic groups are not available. These variables may be of utter interest for future studies since mentalizing, loneliness, pain-related depressive symptoms, and pain may have different patterns in different age or sex groups. Due to sample size limitations, we did not control for potential confounders such as age, gender, and disease duration within the SEM. Future studies with larger samples should include these variables to examine their moderating or mediating effects.

## 5. Conclusions

In conclusion, our results contribute to a growing body of evidence suggesting that psychosocial mechanisms—particularly the ability to mentalize and process emotional experiences—may influence pain perception in patients with rheumatic diseases. These findings call for a broader, integrative approach in clinical care, where improving mentalizing capacities and mitigating loneliness and depression could enhance patient outcomes [69]. Future research should employ longitudinal and experimental designs to further investigate the causal pathways and evaluate the efficacy of mentalization-based interventions in clinical settings.

## Figures and Tables

**Table 1:** Sociodemographic and clinical characteristics.

	*n*	%
Sex		
Male	15	19.7%
Female	61	80.3%
Age (M = 45.6; SD = 12.5)		
<40	14	18.4%
40–60	49	64.5%
>60	13	17.1%
Smoker		
No	49	64.5%
Yes	16	21.1%
Ex-Smoker	11	14.5%
BMI		
Underweight (BMI ≤ 18.5)	1	1.3%
Average weight (BMI 18.5–25)	29	38.2%
Obese (BMI > 25)	39	51.3%
Missing	7	9.2%
Diagnosis		
Rheumatoid arthritis (RA)	25	32.9%
Axial spondylarthritis (axSpA)	24	31.6%
Peripheral spondylarthritis (pSpA)/ Psoriatic arthritis (PsA)	16	21.1%
Vasculitis/collagenosis	9	11.8%
Others	2	2.6%

**Table 2:** Correlations between mentalizing, loneliness, pain-related depressive symptoms, and pain severity at baseline (T1).

	Loneliness		Depression		ADL Impairment		Leisure Time Impairment		Working Impairment	
	r	(*p*)	r	(*p*)	r	(*p*)	r	(*p*)	r	(*p*)
Mentalizing	−0.38	(<0.001)	−0.57	(<0.001)	−0.39	(<0.001)	−0.43	(<0.001)	−0.44	(<0.001)
Loneliness	--	--	0.23	(0.044)	0.29	(0.013)	0.34	(0.003)	0.33	(0.003)
Depression	--	--	--	--	0.51	(<0.001)	0.54	(<0.001)	0.62	(<0.001)
ADL impairment	--	--	--	--	--	--	0.68	(<0.001)	0.84	(<0.001)
Leisure time impairment	--	--	--	--	--	--	--	--	0.80	(<0.001)
Working impairment	--	--	--	--	--	--	--	--	--	--

r = Pearson correlation coefficient; ADL = activities of daily living.

## Data Availability

The datasets analyzed in this manuscript are not publicly available due to ethical and legal restrictions. Requests for access to anonymized datasets should be directed to the corresponding author (david.riedl@i-med.ac.at).
